# The Role of Autotaxin and LPA Signaling in Embryonic Development, Pathophysiology and Cancer

**DOI:** 10.3390/ijms24098325

**Published:** 2023-05-05

**Authors:** Christiana Magkrioti, Eleanna Kaffe, Vassilis Aidinis

**Affiliations:** 1 Institute for Fundamental Biomedical Research, Biomedical Sciences Research Center Alexander Fleming, 16672 Athens, Greece; magkrioti@fleming.gr (C.M.); kaffe@fleming.gr (E.K.); 2 Department of Immunobiology, Yale University School of Medicine, New Haven, CT 06520, USA

## 1. Introduction

Autotaxin (ATX) or Ectonucleotide Pyrophosphatase/Phosphodiesterase 2 (ENPP2) is a secreted enzyme with lysophospholipase D activity, with its primary function being the extracellular hydrolysis of lysophosphatidylcholine (LPC) to lysophosphatidic acid (LPA), a bioactive lipid. Genetic deletion of ATX results in embryonic lethality due to vascular and neural tube defects suggesting a significant role of ATX in development [1]. In adult life, it is widely expressed with the highest mRNA expression noted in the adipose tissue, the central nervous system and the reproductive organs. At the same time, it is overexpressed upon chronic inflammatory and fibroproliferative disorders such as arthritis, hepatic, pulmonary and kidney fibrosis, as well as cancer [1]. The plethora of ATX reported functions are attributed to the extracellular formation of its product, LPA, a pleiotropic growth factor-like phospholipid, signaling via its G-protein coupled receptors (LPAR1-6) and activating a multitude of cellular signal transduction pathways [1]. Overall, any ATX effect will rely on its local concentration and the availability of LPC, as well as on the expression profile of LPA receptors that mediate LPA signaling and lipid phospholipases (LPPs) that catabolize LPA.

This editorial discusses the latest developments in embryonic development, inflammatory diseases and cancer pathophysiology, as published in the Special *IJMS* Issue on “The Role of Autotaxin and LPA Signaling in Embryonic Development, Pathophysiology and Cancer”, and further discusses seminal articles that were published during recent years.

## 2. Development

Homozygous, ubiquitous ATX knockout mice die at embryonic day 9.5, characterized by vascular defects, neural tube defects, asymmetric headfolds and aberrant chorio-allantoic fusion [2,3,4]. Neurite outgrowth is also compromised upon ATX KO, while exogenous LPA can rescue this phenotype, attributing to ATX and LPA a role in nervous system development [3]. Moreover, a role in vasculature formation is also apparent as early blood vessels fail to develop into mature vessels [4]. Interestingly, overexpression of ATX again led to embryonic death at around E9.5 due to severe vascular defects, which implies that LPA levels need to be tightly regulated [5]. Moreover, a major role for ATX expression was shown in glial fibrillary acidic protein-positive (GFAP^+^) cells (mostly astrocytes) in embryonic development as genetic deletion of ATX specifically from these cells resulted in embryonic lethality urging further dissection [6]. Apart from LPA-mediated effects, ATX seems also to have a non-LPA-mediated impact on embryonic development, such as effects on the maturation of oligodendrocytes [7] and the positioning and adhesion of neuronal progenitors in the developing cortex [8].

Genetic deletion of *Lpar1* results in approximately 50% neonatal lethality with impaired suckling in surviving neonatal pups [9]. Survivors also show reduced size, craniofacial dysmorphism and increased apoptosis in sciatic nerve Schwann cells. The perinatal death and the stunted growth among *Lpar1*^−/−^ neonates are attributed to the suckling defect, possibly due to developmental aberrancies in the olfactory bulb and/or the cerebral cortex leading to defective olfaction [9]. During colony expansion of the *Lpar1*-null mutant, a fully viable variant arose spontaneously, which was characterized by reduced neurogenic ventricular zone, altered neuronal markers, and cortical cell death supporting a role for LPAR1 in normal cortical development [10]. A role for LPA in brain formation was further substantiated by a study that showed that extracellular LPA increased the width and the folding of cerebral cortices ex vivo [11]. Moreover, LPA signaling modulated the cleavage plane orientation of apical neural progenitor cells in vivo, resulting in increased aneuploidy in dividing cells, thus generating somatic genomic mosaicism [12]. Therefore, it is indicated that these stable genomic changes during brain development and cortical neurogenesis are not purely stochastic; rather, they are influenced by extracellular signals, such as LPA [12]. Early LPA signaling is important for brain development, whereas its disruption may lead to neurological and psychiatric disorders.

Homozygous *Lpar2* KO mice are viable and display no apparent phenotypic abnormalities [13]. Regarding *Lpar4* KO mice, a subset dies before weaning, with studies indicating hemorrhage of various organs and dilation of the lymph sacs in the KO embryos [14]. *Lpar6*-KO mice are viable and healthy, whereas *Lpar4;Lpar6*-DKO are embryonically lethal by E11.5, suggesting that the cooperation of the two receptors is necessary for embryonic development [15]. Lethality was due to global vascular deficiencies indicating that LPAR4 and LPAR6 are regulators of angiogenesis [15].

The ATX/LPA/LPAR1 axis is critical for satellite cell differentiation and skeletal muscle regeneration as it is extensively activated upon myoblast to myotube differentiation in vitro and upon skeletal muscle regeneration in vivo [16]. Conditional deletion of *Enpp2* in satellite cells led to smaller myofiber regeneration in an experimental muscle injury model [16]. In agreement with this, ATX overexpressing mice presented muscle hypertrophy and accelerated recovery post-injury. The critical receptor in myogenesis is LPAR1, as shown with an LPAR1 conditional KO recently made [16]. A conditional *Lpar1* KO was also created from another group, which helped identify the neural cell types mediating neuropathic pain [17].

Furthermore, *Lpar1* deletion leads to deformities and impairment of cartilage formation as the ATX/LPA/LPAR1 axis controls the formation of the growth plate and chondrocyte activity [18]. LPA/LPAR1 signaling is mandatory for osteoclastogenesis and osteoclast resorption activity in vitro and in vivo, while LPAR1 and LPAR4 are important in osteoblastic differentiation [18]. *Lpar1*^ΔOb^ mutants (conditional knockout mice for *Lpar1* targeted in early osteoblast precursor cells) are characterized by reduced bone mineralization, cortical thickness and increased bone porosity. On the contrary, murine lines lacking *Lpar4* show a high bone mass phenotype indicating a negative role for LPAR4 in bone morphogenesis. In fact, LPAR1 and LPAR4 seem to play opposing roles during osteogenesis and the commitment of bone marrow-derived mesenchymal stromal (stem) cells (BMSCs) to early osteoblastic or adipogenic lineages, with LPA favoring activation of osteoblast differentiation through LPAR1 and inhibition through LPAR4 [18].

In conclusion, during embryogenesis, the ATX/LPA axis is implicated in brain development, neural tube formation, angiogenesis, myogenesis and bone and cartilage development.

## 3. Pathophysiology

For a considerable span of time, ATX has been implicated in rheumatoid arthritis (RA) as its levels were found elevated in synovial fluid from RA patients [19]. Conditional genetic deletion of *Enpp2* in mesenchymal cells, including synovial fibroblasts (SFs), resulted in disease attenuation in experimental arthritis [20]. Data support a model where TNF and the inflammatory milieu in joints stimulate ATX expression from SFs leading to their autoactivation and their effector functions via LPA/LPAR1, culminating in the pathogenesis of arthritis [1]. According to other studies, ATX derives from osteoclasts and controls inflammation-induced bone erosion and systemic bone loss [18]. Additionally, mice with global deletion of *Lpar1* presented a strong resistance to bone destruction in an arthritis model [21]. In this issue, Zhao et al. study the connection between Phospholipase A1 member A (PLA1A) or phosphatidylserine-specific phospholipase A1 (PS-PLA1), the enzyme with specific substrate preference for sn-1 fatty acids on phosphatidylserine (PS) or lysophosphatidylserine (lysoPS) [22], and systemic autoimmune rheumatic diseases and find yet another role of ATX/LPA in rheumatic diseases [23]. Compared to gout and the non-rheumatoid disease osteoarthritis, the synovial fluids of RA patients showed a higher concentration of PLA1A [23]. Incubation of fibroblast-like synoviocytes with recombinant PLA1A induced IL-8 secretion and this study suggests that PLA1A cleaves membrane-exposed PS into lysoPS, with the latter being subsequently converted to LPA by ATX [23]. Therefore, it is proposed that the IL-8 induction by PLA1A is mediated through ATX and LPA.

Indeed, LPA was shown to promote the induction of IL-8 in another cell system, namely human proximal tubular epithelial cells. These cells were exposed to LPA and 175 other inflammatory–immunological stimuli, and multiplex ELISA was performed. LPA was found to induce the secretion of CCL2, also known as monocyte chemoattractant protein 1 (MCP-1), CCL3, also known as macrophage inflammatory protein-1α (MIP-1α), IL-6, IL-8, CXCL10 and soluble ICAM1 [24]. Moreover, LPA induced the phosphorylation of transcription factors JUN and CREB1 and signaling proteins IκBα and MEK1. Our results suggested that the JNK/c-JUN, MEK/ERK, NFκB, and CREB pathways are implicated in the production of some of the LPA-mediated secreted factors [24]. Furthermore, upon clustering of the 176 stimuli, LPA clustered with proinflammatory molecules such as IL-1α, IL-1β, IL-17α, TNFα and others, attributing to LPA a proinflammatory role. This becomes of significant importance as LPA levels are risen in kidneys of experimental renal fibrosis [25,26]. Similarly, renal ATX protein levels are also increased in a model of kidney fibrosis [27]. Apart from having a proinflammatory role, ATX was also shown to induce the migration, proliferation and differentiation of renal fibroblasts, thus, having a profibrotic role and worsening the disease outcome [27]. LPA-LPAR1 have a profibrotic role, too, as they regulate the levels of profibrotic molecules, such as transforming growth factor beta (TGFbeta) and connective tissue growth factor (CTGF) [25]. Therefore, we conclude that ATX and LPA have both proinflammatory and profibrotic roles in renal fibrosis and nephropathies in general.

Nikitopoulou et al. find a negative role of the ATX/LPA axis in systemic inflammation employing the LPS model of experimental sepsis [28]. Genetic deletion of *Enpp2*, obligatory or induced, increased the survival of mice subjected to this model. This effect is mediated, at least to some extent, through LPAR1 and LPAR5, not LPAR2. Pertaining to human patients, serum ATX levels were higher when patients entered a sepsis phase compared to the levels upon ICU admission, indicating a role for ATX in sepsis and suggesting ATX/LPA as a therapeutic target in severe, systemic inflammatory disorders.

Another paradigm of the ATX/LPA axis implication in systemic inflammation is an acute liver failure (ALF), a rare inflammatory condition with impaired immune responses. Comparisons between the sera of ALF patients versus healthy controls (HC) showed a decrease in several species of LPC, which can be used as biomarkers of poor prognosis [29]. Simultaneously, plasma ATX and LPA were increased in ALF compared to HCs. After culture of peripheral blood mononuclear cells (PBMCs) with LPA, monocytes showed immunophenotypic changes, such as increased PD-L1 expression and reduced expression of pro-restorative phenotypical monocytic markers MerTK, CD163 and CD155 in samples from both ALF and HCs [29]. The same study showed that the effect of LPA on the regulatory phenotype of monocytes is mediated via an LPAR1/3 mechanism, and thus, these receptors are a target for immunotherapy in ALF [29]. Similar findings occurred in Acute-on-chronic liver failure (ACLF), a complication of cirrhosis characterized by abnormalities in the immune system, impaired host’s antimicrobial responses and increased susceptibility to infections. LPCs were also down-regulated in ACLF and associated with mortality, while plasma and hepatic ATX were upregulated [30]. LPA reduced MerTK and CD163 expression and increased TNFa expression ex vivo in monocytes of HCs and patients with ACLF [30]. Therefore, ATX/LPA pathway interventions could be helpful in reprogramming monocytes in ACLF therapeutically.

Another example of systemic inflammation where ATX plays a role is COVID-19. Nikitopoulou et al. found that CoV-2 infection stimulates *ENPP2* mRNA expression in nasopharyngeal swab samples from COVID-19 patients and ATX protein levels in the serum of ICU COVID-19 patients, the latter correlating with IL-6 levels [31]. IL-6, TNF and IL-1β of the COVID-19 cytokine storm have been suggested to stimulate ATX expression or activity in different cell types [32]. At the same time, in reverse, LPA has been shown to stimulate TNF and IL-6 expression in different contexts [24,33], thus proposing a possible interplay of the ATX/LPA axis with the CoV-2-induced cytokine storm. Moreover, ATX levels correlated with endothelial dysfunction markers, suggesting a role for ATX/LPA in COVID-19-induced vascular dysfunction and negatively affected survival [31]. According to a single cell data analysis of publicly available datasets, the authors identify the cell populations that express ATX in the nasopharyngeal swab (NK cells and monocyte-derived macrophages (MoAM)), blood (plasmacytoid dendritic cells (pDCs)), BALF (pDCs, as well as MoAMs) and lung tissue (arterial, mesothelial and cells of the monocytic lineage) [31]. Remarkably, ATX was found mainly expressed in pDCs, the principal interferon (IFN) type I producing cells, both in the circulation and in BALF. At the same time, it was statistically significantly overexpressed in COVID-19 circulating pDCs compared to pDCs from control subjects [31]. However, the genes upregulated in *ENPP2*-expressing DCs are associated with an immature state of DCs, suggesting that *ENPP2* expression from COVID-19 DCs may delay their maturation. This is following Emo et al., who ascribed a negative role for LPA on DC activation [34]. As circulating and lung pDCs were diminished [35] and IFN type I responses were highly impaired in COVID-19 [36], perhaps ATX is implicated in COVID-19 through dampening pDCs numbers and/or responses. Given the commonalities between COVID-19 and IPF [37], anti-fibrotic therapies have been suggested for COVID-19. As anti-ATX drugs have led to clinical trials against IPF, ATX can also be visualized as a drug target for COVID-19.

Among the main comorbidities of COVID-19 is insulin resistance. The ATX/LPA axis has been incriminated in regulating glucose homeostasis and insulin resistance, suggesting ATX as a possible pathologic link between obesity and COVID-19. According to the review of Jose and Kienesberger, ATX levels are upregulated in obesity, correlating with impaired glucose homeostasis and insulin function [38]. At the same time, most studies also point towards an increase in LPA levels upon obesity in mice models and humans, with a positive correlation with BMI. Interestingly, proinflammatory cytokines, insulin and glucose are all inducers of ATX expression in adipocytes (with adipose tissue being the major producer of ATX in the body) [38]. Therefore, in obesity-driven insulin resistance, inflammation, hyperinsulinemia and hyperglycemia can all promote ATX expression, with the latter further promoting insulin resistance and inflammation, forming a vicious cycle. Not surprisingly, haploinsufficiency or adult-global deletion of the ATX gene leads to decreased body weight and ameliorates glucose and insulin tolerance, showing that the ATX/LPA axis exacerbates adiposity and inflammation and leads to impaired energy homeostasis [38]. Pharmacological inhibition of the axis seems to improve glucose homeostasis, adipose tissue fibrosis and cardiac hypertrophy. Conclusively, the ATX/LPA/LPAR axis is implicated in a wide array of obesity-induced complications ranging from insulin resistance and inflammation to tissue fibrosis, cardiomyopathy and mitochondrial dysfunction.

In recent years, ATX and LPA have also been suggested as a link between obesity/insulin resistance and cardiovascular diseases [39]. Accordingly, ATX was detected in endothelial cells (ECs) covering atherosclerotic lesions [40]. Genetic ablation of the ATX gene in the ECs of ApoE^−/−^ mice fed a high-fat diet for 12 weeks decreased atherosclerosis (the plaque and necrotic core area), accumulation of macrophages, monocyte adhesion and inflammation (CXCL1 expression) [40]. Moreover, in aortic ECs in vitro, ATX mediated the mildly oxidized LDL (low-density lipoprotein)-induced expression of CXCL1 by generating LPA 20:4, 16:0 and 18:1. Thus, a scheme is drawn where LDL is mildly oxidized to moxLDL producing LPC, which serves as a substrate to endothelial ATX giving rise to LPA. The latter induces the expression of CXCL1, which can recruit monocytes, therefore increasing the plaque size and promoting atherosclerosis [40].

Endothelial ATX seems to have an important role not only in atherosclerosis but in stroke, too. In the period following a stroke, glutamate release from damaged cells leads to secondary injury in the stroke penumbra (the part of an ischemic stroke that is at risk of progressing to infarction but is still salvageable if reperfused). Therefore, preserving the penumbra is of critical importance for improving the stroke outcome. Upon experimental stroke, plasma ATX and LPA levels were elevated. At the same time, EC-specific ATX deletion reduced them, reduced the infarct size, neuronal loss and inflammation in the penumbral region, as well as significantly improved perfusion in the mouse brain tissue and decreased vascular permeability (which could lead to hemorrhagic transformation) [41,42]. Similarly, ATX and LPAR inhibitors attenuated the blood–brain-barrier (BBB) permeability, which is one of the main problems after stroke, and reduced infarct size [42,43]. In a seminal study, Bitar et al. detected long-lasting increases in brain ATX concentration after stroke in a mouse model and increased cerebrospinal fluid ATX in humans up to 14 days after stroke [44]. Genetic ablation of the ATX gene in astrocytes or pharmacological inhibition of ATX at different time points after experimental stroke inhibited LPA-related cortical excitability, protected the stroke penumbra and improved stroke outcome [44]. Therefore, employing ATX inhibitors in the clinic upon stroke could potentially improve the stroke outcome.

Apart from stroke, ATX is also implicated in aneurysm. Specifically, heterozygous ablation of the RNA methyltransferase NSun2 (NOP2/Sun domain family, member 2) significantly reduced experimental abdominal aortic aneurysm in mice by downregulating ATX protein expression in the area of the injury [45]. Furthermore, ATX, which was shown to be located on the endothelium, induced T cell adhesion on ECs by mediating the induction of integrins on T cells, binding on them and activating the FAK/Src pathway [45]. Therefore, it is concluded that NSun2 promotes vascular inflammation and aneurysm formation by increasing T-cell recruitment through upregulation of ATX. The latter probably occurs through NSun2-mediated methylation of ATX mRNA [46]. Given the pleiotropic effects of ATX/LPA on T cells depending on the mediating LPAR, further studies are needed to decipher which LPA receptors are responsible for T cell recruitment in the area of the aneurysm.

Collectively, ATX seems to have a detrimental role both in systemic inflammation in the form of sepsis, COVID-19, liver failure and obesity and in organ-specific inflammatory pathologies such as RA, kidney fibrosis, atherosclerosis, stroke and aneurysm. Inhibitors of the ATX/LPA/LPARs pathway could be beneficial in the above contexts.

## 4. Cancer

Another ATX-related pathology that this Special Issue examines is cancer. Panagopoulou et al. publicly compared available methylomes of malignant and the respective benign tissues from seven different cancer types [47]. Healthy tissues were methylated in all *ΕNPP2* gene body CGs but had lower methylation levels in Promoter Associated (PA) regions. In the majority of the cancers examined, all the promoter- or 1st exon-associated CGs, regions important for transcriptional regulation, were differentially methylated CpGs (DMCs), and specifically hypermethylated, across different cancer types. In contrast, CGs in the gene body region were hypomethylated. Altered methylation is likely to affect *ENPP2* transcription as 4 CGs found in the *ENPP2* promoter and identified as DMCs in most malignancies examined are located in transcription factor binding sites. These results point towards downregulation of *ENPP2* in most cancer types examined. *ENPP2* methylation and expression analysis in prostate cancer (PC) datasets showed that increased methylation in PA regions correlates with a decreased expression of *ENPP2* in PC. The same was also valid for lung cancer. On the contrary, hepatocellular carcinoma (HCC) showed increased ATX expression levels compared to normal tissues, despite the upregulation of methylation in all the TSS and 1st exon-related CGs, thus proposing a methylation-independent regulation of *ENPP2* in HCC. However, in all three types of cancer, increased methylation of several CGs was correlated with poor tumor parameters, and, therefore, *ENPP2* methylation of specific CGs could be employed as a biomarker of cancer progression.

In the second paper of Panagopoulou et al. in this special edition [48], 10 DMCs were detected between breast cancer (BrCa) patients and healthy individuals. Among them, the promoter-associated DMCs were hypermethylated in BrCa in relation to control tissues. Expression analysis showed downregulation of *ENPP2* expression in BrCa; in fact, methylation of promoter-associated CGs was inversely correlated to the expression of *ENPP2*. Differential methylation analysis also indicated an involvement of *ENPP2* methylation in the BrCa early carcinogenic process and metastasis, but not in the progression between stage I/II and stage III BrCa. Moreover, observation of methylation in circulating cell-free DNA (ccfDNA) revealed more frequent methylation in ccfDNA of BrCa patients than in healthy individuals [48]. In fact, methylation correlated with the presence of multiple metastatic sites. Conclusively, the *ENPP2* gene is methylated and lower expressed in BrCa compared to normal tissue suggesting methylation as a regulatory mechanism in BrCa. This is in accordance with a previous study showing that inflammatory mediators from breast tumors stimulate ATX secretion from adjacent adipose tissue rather than the breast cancer cells per se [49]. Furthermore, the authors suggest the first exon methylation as a putative BrCa biomarker which can be assessed from a liquid biopsy.

Yang et al., in their paper [50], study breast cancer as well but from another perspective. They focus on human cytomegalovirus (HCMV), whose DNA and/or proteins are highly detected in breast tumors and are potentially associated with invasiveness and metastasis. Specifically, they study the susceptibility of BrCa cells and fibroblasts to HCMV infection and find better infectivity in breast fibroblasts compared to BrCa cell lines [50]. Furthermore, infection rates of the different cell lines correlated with the expression of platelet-derived growth factor receptor-α (PDGFRα), a receptor that facilitates HCMV entry. HCMV infection of fibroblasts stimulated the secretion of proinflammatory factors, whereas it decreased the secretion of inflammatory factors in infected BrCa cells. However, it mildly increased the ATX mRNA levels in both cell types, indicating a possible role for ATX/LPA in viral-induced BrCa development. Conclusively, HCMV infection preferentially targets tumor-associated fibroblasts, or BrCa cells expressing PDGFRα, mainly affecting the tumor microenvironment rather than cancer cells, activating both inflammation and the ATX/LPA axis that could enhance metastasis [50]. The role of the ATX/LPA axis on BrCa cells was also tested in a study that compared the effect of LPA on different types of BrCa cell lines (triple negative, luminal A, luminal B and HER2+ cells), which found that LPA has a stimulatory effect on cellular functions (proliferation, migration, invasion) of triple-negative and luminal A BrCa cell lines [51] and on IL-8, IL-6 and TNF-alpha secretion in triple negative cells [51]. The LPAR expression profile of each cell line will determine the final effect of LPA. Conclusively, patients with specific subtypes of BrCa may benefit from an anti-ATX/LPA therapy, but further studies are needed to establish this statement.

Despite the results mentioned above about hypermethylation and consequent downregulation of *ENPP2* in several cancer types, it is well known that ATX-LPA is overproduced in various types of cancer. In ovarian cancer, for example, LPA is overproduced and has an established role as a potent mitogen that stimulates tumor cell proliferation, migration and invasiveness. However, whether the ATX/LPA pathway facilitates malignant progression and resistance to therapy through anti-tumor immunity suppression remains largely unexplored. In their study, Chae et al. report that LPA operates as a negative regulator of type-I IFN responses, which are required for robust anti-cancer immune responses and immunotherapy’s optimal efficacy [52]. Specifically, they found that genetic ablation of *Enpp2*/ATX in ovarian cancer cells (used for an orthotopic ovarian cancer model) diminished malignant ascites development and markedly extended overall host survival. Moreover, LPA exposure of several types of mouse and human dendritic cells inhibited the production of IFNβ, and this occurs through a prostaglandin E2 (PGE_2_) mechanism. Finally, it is shown that ATX deficiency drastically improves the therapeutic effects of drugs that elicit type-I IFN responses [52]. Similarly, Matas-Rico et al. showed that ATX secreted by melanoma cells or LPA is chemorepulsive for patient-derived tumor-infiltrating lymphocytes and healthy blood-derived CD8^+^ T cells ex vivo, impairing tumor regression [53]. Moreover, ATX expression by tumors in vivo impaired the infiltration of CD8^+^ T cells from the blood, without affecting the infiltration of conventional CD4^+^ T cells and Treg cells into the tumor [53]. This chemorepulsion seems to occur through LPAR6, whereas the well-known ATX-mediated T cell motility that enhances their transmigration into secondary lymphoid organs is positively regulated by LPAR2 [54,55]. As efficient infiltration of T cells into tumors determines the response to immunotherapies, these data put ATX/LPA in the center of tumor immunity in the tumor microenvironment. Concerning the source of tumor ATX, single cell analysis in patient melanoma tumors showed that ATX is expressed not only by malignant cells but also cancer-associated fibroblasts, macrophages and endothelial cells, suggesting that tumor ATX can derive from many sources [53]. Therefore, even in the case of *ENPP2* hypermethylation in malignant cells, total tumor ATX levels can be elevated via its secretion from non-malignant stromal cells. The ATX/LPA axis then creates a T cell-excluding, pro-tumorigenic microenvironment that promotes metastasis; thus, the targeting of the ATX/LPA axis in cancer is of therapeutic potential.

## 5. Conclusions

In conclusion, the ATX/LPA axis is active during development and is re-activated in several pathologies ranging from low-grade inflammatory conditions such as obesity to high-grade inflammatory conditions such as COVID-19, acute liver failure and sepsis. Furthermore, it remains a target for treating chronic inflammatory and fibroproliferative diseases such as RA and fibrosis, as well as cancer. At the same time, it has been assigned a role in atherosclerotic diseases, stroke and aneurysms, too. Therefore, inhibitors against ATX or LPARs could have great potential in attenuating the aforementioned pathologies.
